# Global Visual–Inertial Localization for Autonomous Vehicles with Pre-Built Map

**DOI:** 10.3390/s23094510

**Published:** 2023-05-05

**Authors:** Yun Hao, Jiacheng Liu, Yuzhen Liu, Xinyuan Liu, Ziyang Meng, Fei Xing

**Affiliations:** 1 Department of Precision Instrument, Tsinghua University, Beijing 100084, China; 2 Robotics X, Tencent, Shenzhen 518057, China

**Keywords:** visual–inertial localization, state estimation, pre-built map

## Abstract

Accurate, robust and drift-free global pose estimation is a fundamental problem for autonomous vehicles. In this work, we propose a global drift-free map-based localization method for estimating the global poses of autonomous vehicles that integrates visual–inertial odometry and global localization with respect to a pre-built map. In contrast to previous work on visual–inertial localization, the global pre-built map provides global information to eliminate drift and assists in obtaining the global pose. Additionally, in order to ensure the local odometry frame and the global map frame can be aligned accurately, we augment the transformation between these two frames into the state vector and use a global pose-graph optimization for online estimation. Extensive evaluations on public datasets and real-world experiments demonstrate the effectiveness of the proposed method. The proposed method can provide accurate global pose-estimation results in different scenarios. The experimental results are compared against the mainstream map-based localization method, revealing that the proposed approach is more accurate and consistent than other methods.

## 1. Introduction

Accurate localization represents a fundamental capability for autonomous vehicles. Recently, vision-inertial odometry (VIO) solutions [1,2,3,4,5] have advanced significantly in this area due to their low cost and high efficiency. The vehicle’s 6 DOF pose is estimated by tracking landmarks through consecutive camera frames and integrating inertial measurement unit (IMU) measurements. However, the performance of these VIO systems is limited by drift during long-term simultaneous localization and mapping (SLAM) operations. To achieve a more accurate and robust localization, the fusion of information from VIO and global information such as global navigation satellite system (GNSS) has received considerable attention [6,7,8,9]. However, GNSS-based localization is not accurate enough for vehicles to complete some significant tasks, such as defense and security applications. Meanwhile, GNSS is typically not available indoors. Therefore, GNSS is not reliable enough to be employed in critical situations.

Another solution for obtaining drift-free poses is to match observations of the vehicle to a pre-built map [10,11]. Such a map is constructed with the VIO system, generally containing numerous keyframes and point features. This approach has several advantages such as the provision of map reference coordinates, the potential for reuse in subsequent tasks and the capability to compensate for drift over long travel distances. However, previous studies of map-based localization have not addressed the issue of obtaining a global pose of the vehicle. Without global sensor measurements in the map, map-based localization methods only obtain the vehicle’s pose relative to the local map reference frame rather than the more generic global coordinates. Furthermore, the VIO system’s localization drift may result in accumulated errors that are registered with the pre-built map, potentially leading to inaccurate estimates. Once the pre-built map is wrong, map-based measurements are no longer reliable.

In order to fuse the pose from VIO and the pose from map-based localization, the relative transformation between the odometry reference frame and map reference frame must be aligned. Most studies in the field of map-based localization assume the transformation between the odometry frame and map frame to be constant. However, the transformation cannot be accurately observed. It should be estimated in real-time. By neglecting this issue, map-based localization processes blindly trust the map, resulting in overestimation and lower accuracy.

In this paper, we propose a two-stage global drift-free map-based localization framework to estimate the global pose of the vehicle. During the offline keyframe-based map-building process, we use a GNSS-aided VIO system to build an accurate and reliable global map. All sensing data (visual, inertial and GNSS) are collected to construct the map, which consists of keyframes as well as feature points with their corresponding descriptors. Later, during the global drift-free localization process, an optimization-based VIO is executed to track the vehicle’s pose. The camera frame is registered to the map by extracting and tracking the same type of image features as in the map. These map-based measurements, along with measurements of egomotion of the vehicle based on VIO, are then fused in a pose-graph optimization. The global pose of the vehicle as well as the transformation between the local odometry reference frame and global map reference frame are estimated jointly. Finally, the consecutive global drift-free pose can be obtained. The contributions of this work are as follows:

- We propose a two-stage global drift-free map-based localization framework. In the first stage, we construct a keyframe-based global map. In contrast to the existing studies [5,10,12], the proposed method tightly fuses global measurements and visual and inertial information, providing the absolute pose to the keyframes, thereby decreasing the map error. In the second stage, with the pre-built map, the localization algorithm aligns the VIO output with map-based measurements to obtain the global drift-free pose.
- The transformation between the local reference frame and global map reference frame is introduced into the state vector for online estimation by using a global pose-graph optimization, such that these two frames can always be accurately aligned, further improving the localization accuracy and consistency.
- The proposed framework has been evaluated on both public datasets and real-world scenes. The results demonstrate that robust, high-precision localization can be achieved.

The remaining part of the paper proceeds as follows. Section 2 discusses the related work. Section 3 is concerned with the methodology used for the two-stage global drift-free map-based localization framework. Section 4 introduces the datasets employed in the experiments. In Section 5, the experimental results are presented and the conclusions and discussions are in Section 6.

## 2. Related Works

### 2.1. Visual–Inertial Odometry

As a result of the complementary characteristics between the IMU and camera, VIO has gained substantial research interest as a viable solution for the localization problem of autonomous vehicles. In early work, Weiss et al. [13] proposed an extended Kalman filter (EKF) framework to build a VIO system via the fusion of measurements obtained from both IMU and camera. This method directly fuses the measurements from sensors and the outputs from the sensors are independent and decoupled. In recent years, several artificial-intelligence-based methodologies have been proposed for fusing visual–inertial data to estimate the poses of unmanned aerial vehicles (UAVs). Notably, VIIONet [14] and HVIOnet [15] leverage an end-to-end deep learning architecture to achieve this objective. In general, the fusion of visual and inertial information can be divided into tightly coupled and loosely coupled methods. In this paper, we focus on tightly coupled VIO methods, as they have been shown to be more accurate than loosely coupled methods and have already been widely applied. The mainstream tightly coupled VIO methods can be categorized into two principal groups: filter-based algorithms and optimization-based algorithms.

Jones et al. [16] estimated the camera pose and map landmarks simultaneously using EKF. However, as time expands, the number of landmarks increases exponentially, leading to a substantial growth in the state dimension that presents significant computational challenges. To improve the computational efficiency, Mourikis et al. [1] added only the camera states in the sliding window to the state and ignored the landmarks, resulting in a fast and light-weight system. Later, Li et al. [2] analyzed the observability of MSCKF and enhanced the consistency of the system. However, a drawback of this method is that the update step is carried out when the features exit the camera’s field of view, resulting in only partial utilization of the current measurements in the filter. Bloesch et al. [17] proposed a robust visual–inertial odometry, with a new representation of the landmarks. However, there is a theoretical limitation for filter-based algorithms: nonlinear measurements must be linearized before processing, which may introduce large linearization errors into the estimator and influence the quality of the estimation. Moreover, the linearization errors can cause the filter to be inconsistent [18]. In general, optimization-based algorithms outperform filter-based algorithms in terms of accuracy and consistency because re-linearization is performed during each iteration step. Even though this operation requires additional computation, most devices can run optimization-based algorithms in real time.

Optimization-based algorithms formalize the measurements from sensors as a graph, which are then optimized using iterative algorithms. Leutenegger et al. [4] proposed a keyframe-based optimization method that nonlinearly optimizes the error of vision and IMU jointly for pose estimation. VINS-Mono, an excellent algorithm proposed in [5], can perform initialization, relocalization, loop-closure detection and other functions. Nevertheless, drift from VIO is inevitable due to sensor noise and modelling errors. Moreover, drift accumulates in a large-scale environment, leading to incorrect pose estimation. Despite the fact that the majority of VIO methods contain a loop-closure-detection module that can eliminate drift by recognizing the places already visited by the vehicle, the module will fail for trajectories without loops. On the other hand, VIO methods are local pose-estimation methods in essence, which means that they can only estimate the pose in the local frame (generally the first camera frame). The trajectories will be different when the vehicle starts from different start points even in the same environment. The previous information cannot be directly used due to the lack of a unified global coordinate system.

### 2.2. Fusion of Visual–Inertial Odometry and Global Positional Measurements

Global measurements, such as GNSS, can be used to reduce the drift from VIO effectively. The fusion of global measurements with VIO can be classified into two primary types: loosely coupled and tightly coupled. The former fuse the global measurements and states of VIO directly. The typical representative is VINS-Fusion [6]. In this approach, an independent VIO system is utilized to provide pose estimates that are subsequently fused with GNSS measurements through a pose-graph optimization. Mascaro et al. [7] add an additional node to the multi-sensor fusion pose-graph optimization, which serves to constrain the absolute orientation. Yan et al. [19] proposed a robust adaptive Kalman filter to improve the performance of the integration of GNSS, IMU and image. However, these loosely coupled approaches fail to fully leverage the available sensor data.

In the tightly coupled approaches, all the measurements from the global sensor, camera and IMU are used for state estimation. Li et al. [8] proposed a tightly coupled multi-GNSS/IMU/vision integration model based on EKF. The experimental results on tree-lined roads demonstrated that this method effectively enhances the positioning accuracy and continuity in GNSS-constrained environments. Lee et al. [20] proposed a method for the online spatiotemporal calibration of a GPS-aided VIO system. The system fuses intermittent GPS measurements as well as camera and IMU measurements through the MSCKF framework. For optimization-based frameworks, Cioffi et al. [21] proposed a method for formulating global position residuals by leveraging the IMU preintegration. This method tightly fuses global position measurements and vision with IMU measurements through nonlinear optimization, with a negligible increase in computational cost. Furthermore, Liu et al. [22] proposed a method that incorporates GNSS pseudorange residuals and Doppler shift residuals into the cost function within an optimization-based VIO framework. Reference [23] proposed an invariant filter approach to fuse visual, inertial and raw GNSS data. Due to the excellent performance of tightly coupled approaches, we use a tightly coupled optimization framework to implement the map-building process.

### 2.3. Map-Based Visual–Inertial Localization

In addition to introducing global measurements, map-based measurements can eliminate the drift in visual odometry. For example, Zuo et al. [24] presented a visual localization system that employs stereo cameras to localize the vehicle based on an a priori map that had been generated using a 3D LiDAR. Warren et al. [25] proposed a vision-based route-following system that enables UAVs to return safely in the event of GNSS failure. When GNSS is working well, the system uses a visual teach-and-repeat framework to build a map of the environment. In case of GNSS failure, the UAV’s image can be matched with the map for localization, allowing it to return to the take-off location.

Moreover, there are some methods that use the available map with geotags (Google Street View, satellite imagery, etc.) to localize the vehicle. Litman et al. [26] described a method for estimating the position of a UAV in GNSS-denied environments. This method employs pre-saved satellite imagery to aid the VIO system in achieving global localization in environments where GNSS signals are unavailable. Jaenal et al. [27] proposed a method that involves matching the keyframes with the city-scale appearance map with geotags to address drift in the Visual-SLAM trajectory, resulting in a map-aligned trajectory with improved accuracy. However, these methods are limited in their application scenarios.

On the other hand, few studies have focused on VIO-based map-based localization methods. Some of the aforementioned VIO systems can save a map during the first run and load the saved map during the subsequent run. For instance, VINS-Mono [5], VINS-Fusion [6] and Maplab [10] have the ability to save and load maps. Oleynikova et al. [12] proposed a real-time localization system that keeps the VIO frame consistent and corrects drift using map-based measurements while aligning the VIO frame and global map frame. However, all these methods do not build a global map with global measurements. Building a local map is of little use in reducing drift. Surber et al. [28] presented a system that uses VIO to first build a reference map, followed by utilizing geometric image-based localization during subsequent operations in the same area to register the image to the map and estimate the UAV’s pose relative to the map. By using GNSS priors, the system can globally localize on pre-built 3D maps. Our work differs in that we build a global map with keyframes instead of only 3D landmarks. This will improve the efficiency of search and estimate the transformation between the local VIO reference frame and global map reference frame to enhance the consistency. Furthermore, most existing approaches regard the transformation between the global and local frame as a fixed value, which can introduce inconsistencies and errors in the registration results, as the transformation estimation relies solely on the initial alignment outcome. We regard the transformation as an augmented variable introduced into the state in order to utilize map-based measurements and correct the drift from VIO.

## 3. Method

**Figure 1:**
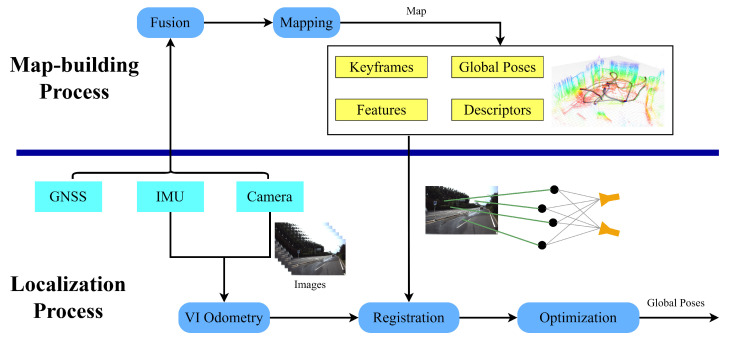
Overview of the two-stage global drift-free map-based localization framework.

In this section, we discuss the details of the proposed two-stage global drift-free localization method. Our framework consists of two parts: an offline keyframe-based map-building process and a global drift-free localization process. An overview of the framework is outlined in Figure 1. Based on the information from an IMU, a camera and a GNSS, a global keyframe-based map that contains keyframes, global poses, features and corresponding descriptors is constructed. Based on the obtained map, the camera frame of the VIO can be registered to the map using an image-matching method and PnP RANSAC algorithm. By relying on a global pose-graph optimization, global poses can be obtained.

### 3.1. Notation

We consider six reference frames in this paper, including the map frame {G}, the local inertial frame {L}, the camera frame {C}, the current IMU (body) frame {I}, the map keyframe frame {K} and the global sensor frame {E}. The map frame {G} is fixed in the world, also called the global frame or world frame. *L* is established at the initial position of the vehicle, serving as the origin of the local VIO estimation. The direction of gravity is aligned with the *z*-axis of frame {L}. The camera frame {C} is attached to the optical center of the camera and {I} is rigidly attached to the vehicle. The transformation between {I} and {C} is usually calibrated beforehand and can be considered known. Therefore, we assume that these two frames are coincident in what follows. The global map contains keyframes, global poses, features and corresponding descriptors. The pose of the map keyframe frame {K} is represented in {G}. The global sensor frame {E} is rigidly attached to {I}. The translation ${}^{I}p_{E}$ can be obtained from the offline calibration. We use the notation ${}^{G}\left(\cdot \right)$ to represent a quantity in the map frame {G}, and corresponding notation for the other frames. We use ${}^{L}T_{I_{k}}=\left[\begin{array}{c} {}^{L}p_{I_{k}}\\ {}^{L}q_{I_{k}} \end{array}\right]$ to denotes the pose of {I} in {L} at time-step *k*, and ${}^{L}v_{I_{k}}$ denotes the velocity of {I} in {L}. The rotation matrix representation is ${}^{L}R_{I_{k}}$. Quaternions and rotation matrices with the same superscript and subscript represent the same rotation. The notation $\hat{\left(\cdot \right)}$ is used to represent the noisy measurement.

**Figure 2:**
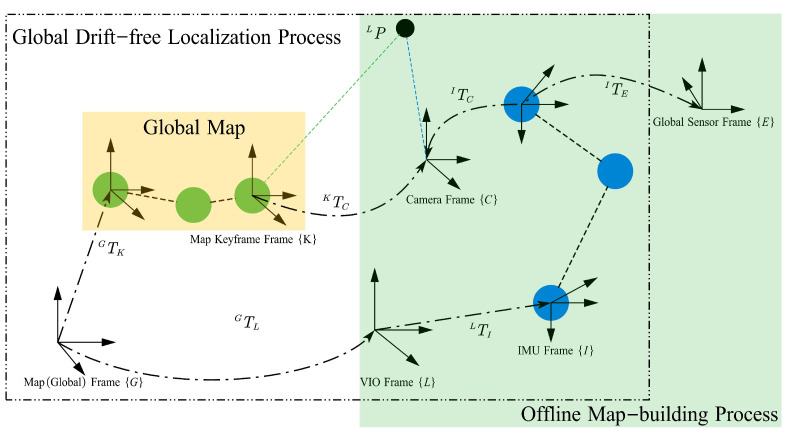
The figure shows the coordinate frames used in this study and the relative transformation between the different coordinate frames. Blue vertexes represent the poses of the IMU frame in the local VIO frame {L}. In the offline keyframe-based map-building process, the global sensor frame {E} represents the pose of the receiver antenna in the case of GNSS measurements. GNSS-aided VIO outputs the global poses of the vehicle, and the pose graph is saved as a keyframe-based global map. In the global drift-free localization process, the global map is composed of the poses of keyframes, which are represented in map frame {G} and indicated by green vertexes. The black circle is the 3D landmark, which is triangulated from keypoint tracks across keyframes in {L}. The 3D landmark observed by the current keyframe and the map keyframe together can be utilized for map-based measurements.

Figure 2 shows the defined coordinate frames and their relative transformations.

### 3.2. Offline Keyframe-Based Map-Building Process

In this part, we describe how to construct a keyframe-based global map. First, the IMU–camera data and global position measurements are fused in a tightly coupled approach. After the pose-graph optimization, we save the pose graph as a global map.

#### 3.2.1. Visual–Inertial Odometry

We provide a brief overview of the VIO that we employ in our method, namely VINS-Mono [5], which is a sliding-window keyframe-based nonlinear optimization framework. As per the definition proposed by [5], the states within a sliding window are defined as follows:

(1) $$\chi_{VIO}=\left[\chi_{I},\chi_{\lambda }\right],$$

where $\chi_{\lambda }$ comprises the inverse depth of the landmarks when first observed in the camera frame and $\chi_{I}=\left[x_{0},x_{1},\cdots ,x_{m}\right]$ comprises *m* IMU states. The *k*-th IMU state is defined as the vehicle’s position ${}^{L}p_{I_{k}}$, velocity ${}^{L}v_{I_{k}}$, orientation quaternion ${}^{L}q_{I_{k}}$ and IMU bias $b_{a},b_{g}$. Therefore, the IMU state representation is

(2) $$x_{k}=\left[{}^{L}p_{I_{k}}{,}^{L}v_{I_{k}}{,}^{L}q_{I_{k}},b_{a},b_{g}\right].$$

The IMU measurements are integrated between two consecutive frames. To avoid the repeated integration of IMU measurements at every optimization step, the IMU preintegration derivation proposed in [18] is used. Given the time interval $\left[i,j\right]$ for two consecutive camera frames at time-steps *i* and *j*, ${}^{L}p_{I}$, ${}^{L}v_{I}$ and ${}^{L}q_{I}$ can be propagated by using the accelerometer and gyroscope measurements. The propagation in the frame $\left\{I_{i}\right\}$ can be expressed as

(3) $$\begin{array}{cc} \; & {}^{I_{i}}R_{L}{}^{L}p_{I_{j}}={\;}^{I_{i}}R_{L}{(}^{L}p_{I_{i}}+{\;}^{L}v_{I_{i}}\Delta t_{i}-\frac{1}{2}{}^{L}g\Delta t_{i}^{2})+{\;}^{I_{i}}{\hat{\alpha }}_{I_{j}},\\ \; & {}^{I_{i}}R_{L}{}^{L}v_{I_{j}}={\;}^{I_{i}}R_{L}{(}^{L}v_{I_{i}}-{}^{L}g\Delta t_{i})+{\;}^{I_{i}}{\hat{\beta }}_{I_{j}},\\ \; & {}^{I_{i}}q_{L}⊗{}^{I_{j}}q_{L}={\;}^{I_{i}}{\hat{\gamma }}_{I_{j}}, \end{array}$$

where ${}^{L}g$ is the gravity vector in frame {L}. ${}^{I_{i}}{\hat{\alpha }}_{I_{j}}$, ${}^{I_{i}}{\hat{\beta }}_{I_{j}}$ and ${}^{I_{i}}{\hat{\gamma }}_{I_{j}}$ are the preintegration terms, which are exclusively determined by the IMU measurements captured between the two frames.

When considering the discrete-time case and assuming IMU measurements are available at time-step *k* within the time interval $\left[i,j\right]$, it is possible to recursively compute the mean values of the IMU preintegration terms α, β, and γ by using the Euler numerical integration method. The expression is as follows:

(4) $$\begin{array}{cc} \; & {}^{I_{i}}{\hat{\alpha }}_{I_{k+1}}={\;}^{I_{i}}{\hat{\alpha }}_{I_{k}}+{\;}^{I_{i}}{\hat{\beta }}_{I_{k}}\delta t+\frac{1}{2}R{(}^{I_{i}}{\hat{\gamma }}_{I_{k}})\left({\hat{a}}_{k}-b_{a_{k}}\right)\delta t^{2},\\ \; & {}^{I_{i}}{\hat{\beta }}_{I_{k+1}}={\;}^{I_{i}}{\hat{\beta }}_{I_{k}}+R{(}^{I_{i}}{\hat{\gamma }}_{I_{k}})\left({\hat{a}}_{k}-b_{a_{k}}\right)\delta t,\\ \; & {}^{I_{i}}{\hat{\gamma }}_{I_{k+1}}={\;}^{I_{i}}{\hat{\gamma }}_{I_{k}}⊗\left[\begin{array}{c} 1\\ \frac{1}{2}\left({\hat{\omega }}_{k}-b_{g_{k}}\right)\delta t \end{array}\right], \end{array}$$

where δt is the time interval between two consecutive IMU measurements. $R{(}^{I_{i}}{\hat{\gamma }}_{I_{k}})$ is the rotation matrix of ${}^{I_{i}}{\hat{\gamma }}_{I_{k}}$. ${}^{I_{i}}\alpha_{I_{i}}={\;}^{I_{i}}\beta_{I_{i}}=0$. ${}^{I_{i}}\gamma_{I_{i}}$ is equal to the identity quaternion.

Within the framework, optical flow algorithms are utilized to detect and track the existing features across consecutive frames for each image. Additionally, new corner features [29] are detected and described using the BRIEF descriptor [30] to ensure a minimum number of features in each image. The selection of keyframes is based on the average parallax distance from the previous keyframes and the quality of tracking. Generally, the classic VIO problem can be described as a joint nonlinear optimization. As proposed in [5], the IMU preintegration is aligned with the vision-only structure to obtain the necessary initial values of velocity, the gravity vector and other parameters. After initialization, the VIO system optimizes the state in a local bundle adjustment. To reduce the computational complexity, only the keyframes are temporarily kept in the sliding window, while the previous keyframes are marginalized out of the window. Therefore, the estimation of the state χ can be calculated by minimizing the cost function as

(5) $$\begin{array}{c} J_{VIO}=\sum_{l,j\in C}{∥e_{C}^{l,j}∥}_{W_{C}^{l,j}}^{2}+\sum_{k\in I}{∥e_{I}^{k}∥}_{W_{I}^{k}}^{2}+{∥e_{P}∥}^{2}, \end{array}$$

where I is the set of all IMU measurements in the sliding window. C is the set of all features that have been observed at least twice in the sliding window. $\left|\right|\cdot {\left|\right|}_{W}$ is the Mahalanobis distance weighted by the covariance W. $e_{I}$ and $e_{C}$ are residuals for the IMU and visual measurements, respectively. To be specific, the inertial residuals $e_{I}$ are formulated using IMU preintegration between two consecutive frames in the sliding window. The visual residuals $e_{C}$ describe the reprojection error by reprojecting the landmark ${}^{L}p_{l}$ into keyframe $K_{j}$ and comparing it against the raw visual measurements ${\hat{z}}_{l,j}$. ${∥e∥}_{P}$ are the marginalization residuals, which contain information about past marginalized states.

#### 3.2.2. Global Measurements

The sliding-window keyframe-based VIO optimizes only a small set of recent states within the window, with past states being marginalized. Therefore, the drift will gradually accumulate in the long-term localization process. Moreover, for the later proposed map-based localization process, we need to save the pose graph with the keyframe vertexes and IMU constraint edges as a map. However, the reference map constructed using only VIO is a local map without global information, which means the map may not be constructed correctly in extreme conditions and lacks universality. In our experience, relying only on VIO results in failure sometimes.

Therefore, our algorithm introduces the global measurements and further defines the states as

(6) $$\begin{array}{c} \chi_{M}=\left[\chi_{VIO},\chi_{T}\right], \end{array}$$

where $\chi_{T}$ represents the transformation between the global frame and VIO frame. The global position residuals can be fused into the framework in a tightly coupled way by adding them to the cost function (5), as

(7) $$\begin{array}{c} J_{M}=J_{VIO}+\sum_{m\in G}{∥e_{G}^{m}∥}_{W_{G}^{m}}^{2}, \end{array}$$

where $e_{G}$ is the global position residuals and $W_{G}$ is the weight matrix. G is the set of all global measurements that have been received in the sliding window.

The global residual for a measurement ${}^{G}{\hat{p}}_{E_{m}}$ at time-step *m* that is in the time interval $\left[i,j\right]$ can be formulated as

(8) $$\begin{array}{cc} e_{G}^{m}=\; & {}^{I_{i}}R_{L}{}^{L}R_{G}\left({}^{G}{\hat{p}}_{E_{m}}-{}^{G}R_{L}{}^{L}R_{I_{m}}{}^{I}p_{E}-{}^{G}p_{L}\right)-\left[{}^{I_{i}}R_{L}\left({}^{L}p_{I_{i}}+{\;}^{L}v_{I_{i}}\Delta t_{i}-\frac{1}{2}{}^{L}g\Delta t_{i}^{2}\right)+{\;}^{I_{i}}{\hat{\alpha }}_{I_{m}}\right], \end{array}$$

with ${}^{L}R_{I_{m}}={}^{L}R_{I_{i}}{}^{I_{i}}{\hat{\gamma }}_{I_{m}}$. ${}^{I_{i}}{\hat{\alpha }}_{I_{m}}$, ${}^{I_{i}}{\hat{\gamma }}_{I_{m}}$ and ${}^{G}{\hat{p}}_{E_{m}}$ are the noisy measurements. The covariance of the global residual in (8) is composed of two components. The first component is the covariance $\Sigma_{g}^{m}$ of the noises from the global measurements. The other is the covariance of the noises from the IMU preintegration mesurements, which is denoted as $W_{I}^{m}$. Therefore, the covariance of the global residual can be derived by

(9) $$\begin{array}{c} W_{G}^{m}={}^{I_{i}}R_{L}{}^{L}R_{G}\Sigma_{g}^{m}{\left({}^{I_{i}}R_{L}{}^{L}R_{G}\right)}^{T}+W_{I}^{m}. \end{array}$$

#### 3.2.3. Global Frame Initialization

For the fusion of global position measurements and IMU–camera data, the global frame and VIO frame must be aligned. Thanks to the IMU measurements, the VIO is able to observe two rotational degrees of freedom, namely roll and pitch. The transformation between the global frame and the VIO frame can be modeled using a minimal set of four degrees of freedom, comprising the three translations (*x*,*y*,*z*) and a single rotation around the *z*-axis with yaw angle ψ in the global frame, while the rotation around the other two axes is set to zero. As described in Section 3.2.2, the transformation is introduced to the states. Hence, it is crucial to initialize the transformation before optimizing it. After having received the first global measurements ${}^{G}{\hat{p}}_{I_{0}}and{}^{G}{\hat{p}}_{I_{1}}$ (at least two) and the first VIO output ${}^{L}{\hat{p}}_{I_{0}}and{}^{L}{\hat{p}}_{I_{1}}$ (closest to the global measurements), respectively, an initialization for the transformation between the global frame and VIO frame can be calculated as

(10) $$\begin{array}{c} R{(}^{G}q_{L})=R\left(\psi \right)=\left[\begin{array}{ccc} cos\psi & -sin\psi & 0\\ sin\psi & cos\psi & 0\\ 0 & 0 & 1 \end{array}\right]\\ {}^{G}p_{L}={}^{G}{\hat{p}}_{I_{1-0}}-R{(}^{G}q_{L}){}^{L}{\hat{p}}_{I_{1-0}}, \end{array}$$

where ${}^{G}{\hat{p}}_{I_{1-0}}={}^{G}{\hat{p}}_{I_{1}}-{}^{G}{\hat{p}}_{I_{0}}$ and ${}^{L}{\hat{p}}_{I_{1-0}}={}^{L}{\hat{p}}_{I_{1}}-{}^{L}{\hat{p}}_{I_{0}}$. R(q) represents the rotation matrix corresponding to the quaternion q. ψ can be calculated from $cos\left(\psi \right)=\frac{{}^{G}{\hat{p}}_{I_{1-0}}\cdot {}^{L}{\hat{p}}_{I_{1-0}}}{{∥}^{G}{\hat{p}}_{I_{1-0}}∥∥{}^{L}{\hat{p}}_{I_{1-0}}∥}$. ${}^{G}p_{L}$ and ${}^{G}q_{L}$ are used as the initialization for the optimization.

After each optimization cycle, we need the transformation to obtain the global pose of the vehicle, which can be expressed as

(11) $$\begin{array}{c} {}^{G}T_{I_{k}}={\;}^{G}T_{L}{}^{L}T_{I_{k}}, \end{array}$$

where ${}^{G}T_{L}$ denotes the optimal transformation.

#### 3.2.4. Global Map Saving

Upon completion of the offline keyframe-based map-building process, we save the pose graph as a global map comprising vertices (poses of keyframes) and edges (constraints), as well as feature points with their corresponding descriptors for each keyframe. In order to reduce the memory consumption, the original images are discarded. More specifically, the information that we preserve for the *i*-th keyframe can be represented as:

(12) $$\begin{array}{c} \left[i,{}^{G}p_{I_{i}},{}^{G}q_{I_{i}},D\left(u,v,des\right)\right], \end{array}$$

where *i* is the frame index. ${}^{G}p_{I_{i}}$ and ${}^{G}q_{I_{i}}$ are the position and rotation, respectively, in the global frame {G}. D(u,v,des) is the feature set. Every feature contains 2D coordinates $\left[u,v\right]$ and their BRIEF descriptor. Therefore, all keyframes containing the feature points and descriptors are added to a database for subsequent registration.

### 3.3. Global Drift-Free Localization Process

In this part, we describe the global drift-free localization algorithm, which leverages a global map to eliminate the drift in VIO and obtain the accurate global pose of the vehicle. This process is divided into two steps: the VIO step and the map-based localization step. The VIO step is as described in Section 3.2.1.

#### 3.3.1. Map-Based Measurements

After the map-building process, the constructed keyframe-based global map can be reused, allowing for more accurate localization. We load the map in the VIO step. To register the current VIO keyframe to the global map, the bag-of-words library DBoW2 [31] is used to detect the similarity of two images. DBoW2 scores the images with a pre-trained dictionary to build a bag-of-words (BoW) vector used for describing the image. Based on the BoW vector, the similarity scores of the current VIO keyframe and map keyframes are compared to return the most similar candidates.

When the candidates are detected, the connection between the VIO keyframe and the candidate in the global map is established using feature matching. A fundamental matrix test with RANSAC is performed to remove the outliers of 2D-matched BRIEF pairs. Then, a RANSAC-based perspective-n-point (PnP) algorithm is used to keep only inlier matches from well-supported hypotheses. These inlier matches are between the 2D keypoints of the candidate and 3D landmarks from the VIO. If the tests are passed, we treat the candidate as a correct matched keyframe in the global map.

The PnP RANSAC algorithm not only provides inlier matches, but also estimates the relative pose between the current VIO keyframe and the matched keyframe in the global map. Considering a keyframe *i* and its corresponding matched keyframe *v*, the map-based measurement is defined as

(13) $$\begin{array}{c} {}^{K_{v}}{\hat{T}}_{I_{i}}=\left[\begin{array}{c} {}^{K_{v}}{\hat{p}}_{I_{i}}\\ {}^{K_{v}}{\hat{q}}_{I_{i}} \end{array}\right]. \end{array}$$

#### 3.3.2. Global Pose-Graph Optimization

Map-based measurements are typically susceptible to noise caused by matching errors. Additionally, these measurements are only accessible when the new image closely resembles a keyframe in the global map. Directly employing these measurements to fix the VIO states could result in a sudden jump in the trajectory due to the intermittent properties of the map-based measurements. To address these issues, we perform a global pose-graph optimization, which provides a more accurate estimate of the poses, while also adding some degree of consistency and smoothness.

**Figure 3:**
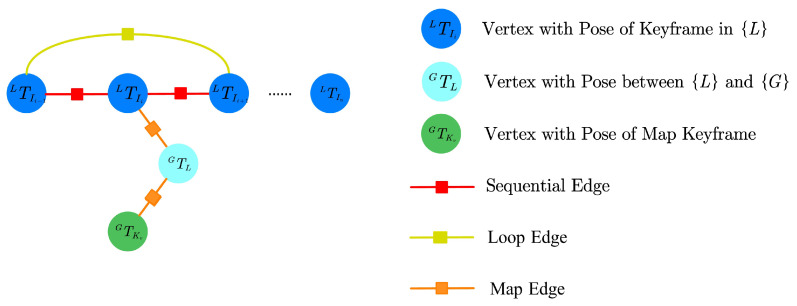
The global pose-graph structure. Every blue vertex represents a pose of a keyframe in {L}, which contains the position and orientation. The red edge between two consecutive blue vertexes is the constraint generated by the VIO step. The yellow edges represent loop edges. ${}^{G}T_{L}$ is the augmented state, connecting the keyframe and global map with map-based measurements. Green vertexes represent the poses of keyframes in the pre-built map, which are fixed in the optimization.

The pose graph contains vertexes and edges. Each vertex in the pose graph represents a pose, which comprises both position and orientation information. The marginalized keyframe in the VIO step is added into the pose graph as a vertex. Moreover, unlike other map-based localization methods that assume the transformation between the global frame {G} of the map and the local frame {L} of the VIO is a fixed value, we augment the transformation ${}^{G}T_{L}$ into the pose graph. Therefore, the definition of the states to be optimized is

(14) $$\begin{array}{c} \chi =\left[{}^{L}p_{I_{0}},{}^{L}q_{I_{0}},\ldots ,{\;}^{L}p_{I_{m}},{}^{L}q_{I_{m}},{\;}^{G}p_{L},{}^{G}q_{L}\right] \end{array}$$

where *n* is the number of poses of all keyframes that are added into the graph. An illustration of the global pose-graph structure is shown in Figure 3.

In the pose graph, there are three types of edges connecting the vertexes, namely sequential edges, loop edges, and map edges. As depicted in Figure 3, a keyframe is connected to its predecessor by a sequential edge. We take advantage of the relative pose between two keyframes to represent the sequential edge, which is determined using VIO directly. Considering a keyframe i+1 and its previous keyframe *i*, the residual of the sequential edge between keyframes i+1 and *i* is defined as

(15) $$\begin{array}{c} e_{S}^{i}{(}^{L}p_{I_{i+1}},{\;}^{L}q_{I_{i+1}},{\;}^{L}p_{I_{i}},{\;}^{L}q_{I_{i}})=\left[\begin{array}{c} {}^{L}R_{I_{i+1}}^{-1}{(}^{L}p_{I_{i}}-{\;}^{L}p_{I_{i+1}})-{\;}^{I_{i+1}}{\hat{p}}_{I_{i}}\\ {}^{I_{i+1}}{\hat{q}}_{I_{i}}{}^{L}q_{I_{i}}^{-1}{}^{L}q_{I_{i+1}} \end{array}\right]. \end{array}$$

The VIO step has a loop-closure module that can detect if a location has been revisited. Upon detection, the keyframe establishes a connection with the loop-closure keyframe via a loop edge in the pose graph, which includes the relative pose between the two frames as determined through relocalization [32]. Similar to the sequential edge, the residual of the loop edge between keyframe *i* and loop-closure keyframe $i^{\prime }$ is defined as follows:

(16) $$\begin{array}{c} e_{L}^{i,i^{\prime }}{(}^{L}p_{I_{i}},{\;}^{L}q_{I_{i}},{\;}^{L}p_{I_{i^{\prime }}},{\;}^{L}q_{I_{i^{\prime }}})=\left[\begin{array}{c} {}^{L}R_{I_{i}}^{-1}{(}^{L}p_{I_{i^{\prime }}}-{\;}^{L}p_{I_{i}})-{\;}^{I_{i}}{\hat{p}}_{I_{i^{\prime }}}\\ {}^{I_{{}^{i}}}{\hat{q}}_{I_{i^{\prime }}}{}^{L}q_{I_{i^{\prime }}}^{-1}{}^{L}q_{I_{i}} \end{array}\right]. \end{array}$$

When map-based measurements are available, the keyframe establishes a connection with the map keyframe via a map edge, which contains the relative position ${}^{K_{v}}\hat{p}I_{i}$ and relative rotation ${}^{K_{v}}\hat{q}I_{i}$ between the frames. Following the aforementioned description, the residual of the map edge between keyframes *i* and *v* can be defined as follows:

(17) $$\begin{array}{c} e_{M}^{i,v}{(}^{L}p_{I_{i}},{\;}^{L}q_{I_{i}},{\;}^{G}q_{K_{v}},\;{}^{G}q_{K_{v}},{\;}^{G}p_{L},{\;}^{G}q_{L})=\left[\begin{array}{c} {}^{G}R_{K_{v}}^{-1}{(}^{G}{R_{L}}^{L}p_{I_{i}}-{\;}^{G}p_{K_{v}}+{\;}^{G}p_{L})-\;{}^{K_{v}}{\hat{p}}_{I_{i}}\\ {}^{K_{v}}{\hat{q}}_{I_{i}}^{-1}{}^{G}q_{K_{v}}^{-1}{}^{G}q_{L}{}^{L}q_{I_{i}} \end{array}\right]. \end{array}$$

Therefore, the final cost function can be formulated as follows:

(18) $$\begin{array}{c} J=\sum_{i,i+1\in S}{∥e_{S}^{i}∥}^{2}+\sum_{i,i^{\prime }\in L}\rho_{L}{∥e_{L}^{i,i^{\prime }}∥}^{2}+\sum_{i,v\in M}\rho_{M}{∥e_{M}^{i,v}∥}^{2}, \end{array}$$

where S is the set of all sequential edges, L is the set of all loop edges and M is the set of all map edges. ρ(·) is the Huber norm to adjust the impact of loops and map-based measurements.

Google Ceres Solver [33] is used for solving this nonlinear problem, which utilizes Levenberg–Marquadt approaches in an iterative way. After each optimization cycle, the optimized ${}^{G}T_{L}$ is updated. Eventually, the global drift-free pose of the vehicle can be calculated as

(19) $$\begin{array}{c} {}^{G}T_{I_{i}}={\;}^{G}T_{L}{}^{L}T_{I_{i}}. \end{array}$$

## 4. Datasets

In this section, we present the datasets employed for evaluating the efficacy of the proposed approach. Specifically, we utilize two publicly available datasets and one proprietary dataset we collected. By conducting experiments on these datasets, we illustrate the effectiveness of the proposed method.

### 4.1. EuRoC Dataset

**Figure 4:**
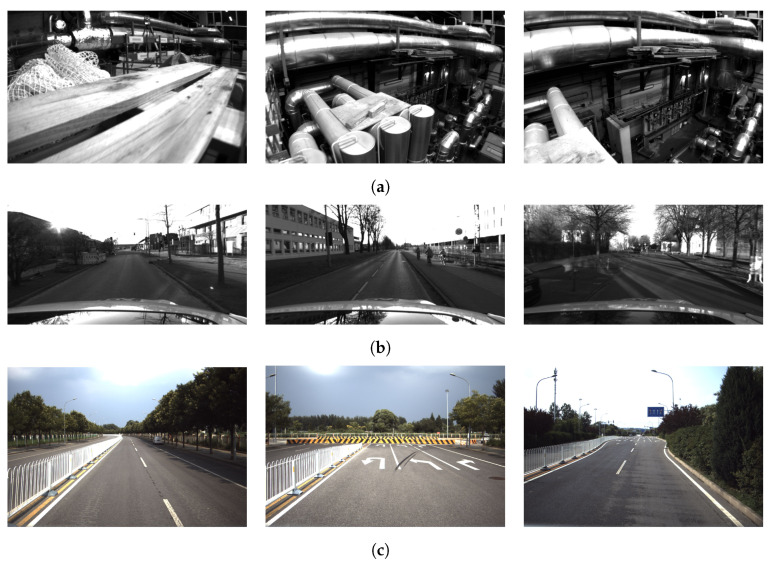
The images sampled from the datasets. EuRoC is a visual–inertial dataset collected onboard a micro aerial vehicle. The 4Seasons and Beiqing Road dataset were collected using vehicles and consist of substantial traffic scenarios recorded with a variety of sensor modalities, including cameras, IMU and GNSS. (**a**) EuRoC dataset. (**b**) 4Seasons dataset. (**c**) Beiqing Road dataset.

The EuRoC dataset [34] is an indoor visual–inertial dataset of a UAV with global position measurements. The dataset contains eleven sequences that were recorded in two different scenes: an industrial hall and an office room. In this study, we focus on the sequences recorded in the industrial hall, which are labeled as MH, to validate the proposed algorithm. Specifically, we use the MH01 sequence to construct a global map, while the MH02–MH05 sequences are employed to perform localization with the map. Figure 4a presents several example pictures of the EuRoC dataset.

### 4.2. 4Seasons Dataset

The 4Seasons dataset [35] offers a comprehensive collection of perceptually challenging seasonal driving scenarios for autonomous vehicles. The dataset consists of more than thirty sequences. The sensors used include stereo cameras, IMU and RTK-GNSS. Additionally, a fused combination of direct stereo visual–inertial odometry and RTK-GNSS technologies yields globally consistent reference poses with centimeter accuracy. In the dataset, we have selected the first two sequences (2020-03-24-17-36-22 and 2020-03-24-17-45-31), which span a distance of roughly 3.8 km and traverse an industrial area within the city. Both sequences present similar lighting and scene characteristics, ensuring successful map matching. Specifically, the first sequence is utilized for map construction, while the second is used for algorithmic testing purposes. The example pictures are shown in Figure 4b.

### 4.3. Beiqing Road Dataset

**Figure 5:**
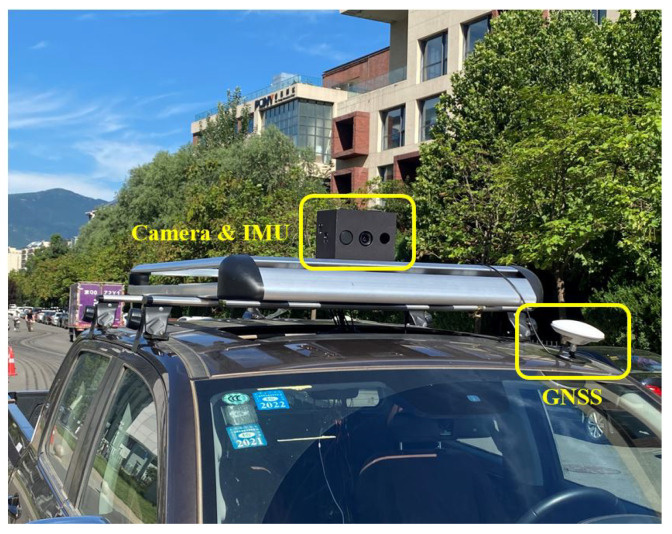
The equipment utilized in the outdoor experiment consists of a global shutter camera (FLIR BFS-U3-31S4C-C) with a resolution of 2048×1536. A time-synchronized IMU (Xsens Mti30-2A8G4) is also integrated into the system. A GNSS antenna is used to receive the signals for global localization in single-point positioning mode. All the sensors are equipped on a pickup truck.

We used a self-developed sensor suit equipped on a pickup truck to collect a real-world dataset in Beijing, which is called the Beiqing Road dataset. Figure 5 illustrates the sensor configuration employed in this study, which comprises a monocular camera (FLIR BFS-U3-31S4C-C), an IMU (Xsens Mti30-2A8G4) and a GNSS (NovAtel OEM718D). The time is synchronized with hardware between the three sensors. The intrinsic parameter of the camera and the extrinsic parameter between the three sensors are calibrated offline. Figure 4c shows a sample of images from the dataset. We collected the data in two trajectories, one for building the map, and the other for testing the algorithms.

## 5. Experiments

We evaluated the proposed method with visual and inertial sensors both on datasets and real-world scenes. The experimental results validate the effectiveness of the proposed method. The implementation of our proposed method is based on the open framework VINS-Mono [5]. All experiments were run on a desktop equipped with a 3.60 GHz Intel Core i7 CPU.

### 5.1. EuRoC Dataset

The EuRoC dataset provides ground-truth position measurements of the recorded sequences in the industrial hall using a Leica Nova MS50 laser tracker. To simulate noisy global position measurements, we intentionally corrupted the ground-truth measurements with zero-mean Gaussian noise during the map-building process. Specifically, we define the Gaussian noise as $N(0,\sigma^{2}\cdot I),\sigma =10\;\mathrm{cm}$.

**Figure 6:**
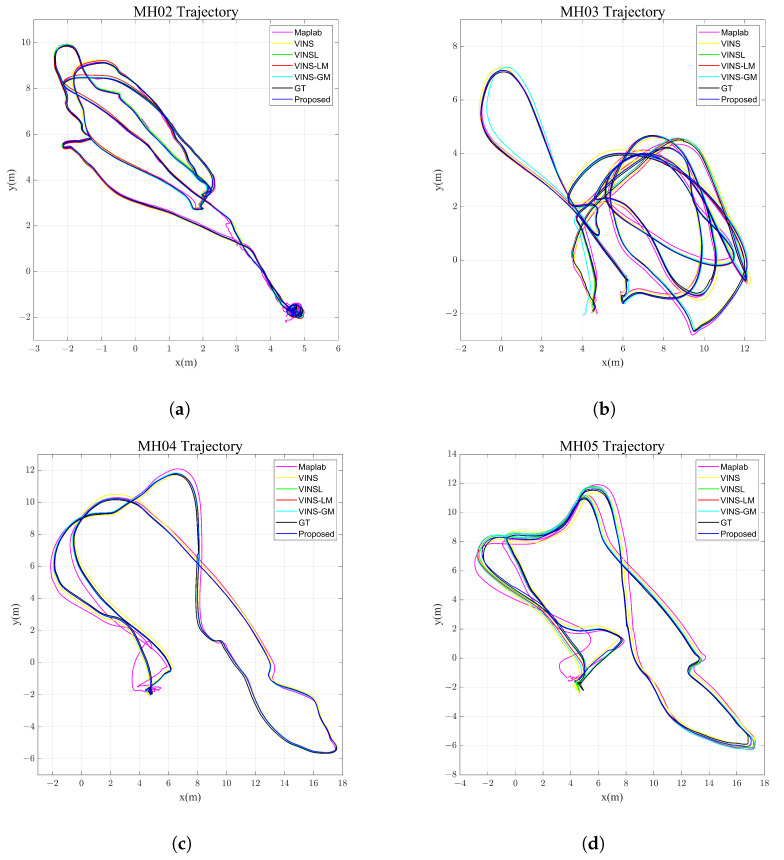
Trajectories of different methods in EuRoC dataset. We compared the results of the different methods, including VINS, VINSL, VINS-LM, VINS-GM and the proposed method. GT represents the ground-truth position of the UAV provided by a Leica Nova MS50 laser tracker and recorded on the base station. (**a**) MH02. (**b**) MH03. (**c**) MH04. (**d**) MH05.

During the experiments, we only utilized the images captured with the left camera. After the map of sequence MH01 was built, we tested six algorithms on the other sequences. We regard VINS-Mono without loop closure as the pure VIO baseline algorithm, which is called VINS (visual–inertial system), and VINSL (visual–inertial system with loop closure) means that VINS can leverage the history information to correct drift when a loop closure is found. VINS-Mono has map-based localization capabilities that use a pre-built local map established using the VINSL or VINS pipeline. We refer to this algorithm as VINS-LM (visual–inertial system with local map). Accordingly, VINS using the global map is called VINS-GM (visual–inertial system with global map). In addition, we also evaluated the performance of Maplab, which is a map-based localization method proposed in [10]. The trajectories of all the sequences are shown in Figure 6.

Table 1 lists the root mean square error (RMSE) of the absolute trajectory error (ATE) calculated for all EuRoC sequences. The results indicate that the proposed algorithm outperforms the other approaches in the majority of the sequences. By leveraging the global map, the map-based measurements can correct the drift of the pose estimation, and global optimization with the augmented state improves the consistency and accuracy of the trajectory. There is a special case in the MH02 sequence where the performance of the proposed algorithm is not the best. This is probably because the global map built using VINS-GM is highly accurate and the map-based measurements provide sufficient constraints. In other circumstances, the results of VIO with a local map are relatively poor. This is due to the absence of global information in the local map and local-map-based measurements providing only limited constraints. Furthermore, Maplab’s inferior performance can be attributed to its filter-based VIO method, which has lower accuracy than optimization-based methods.

**Figure 7:**
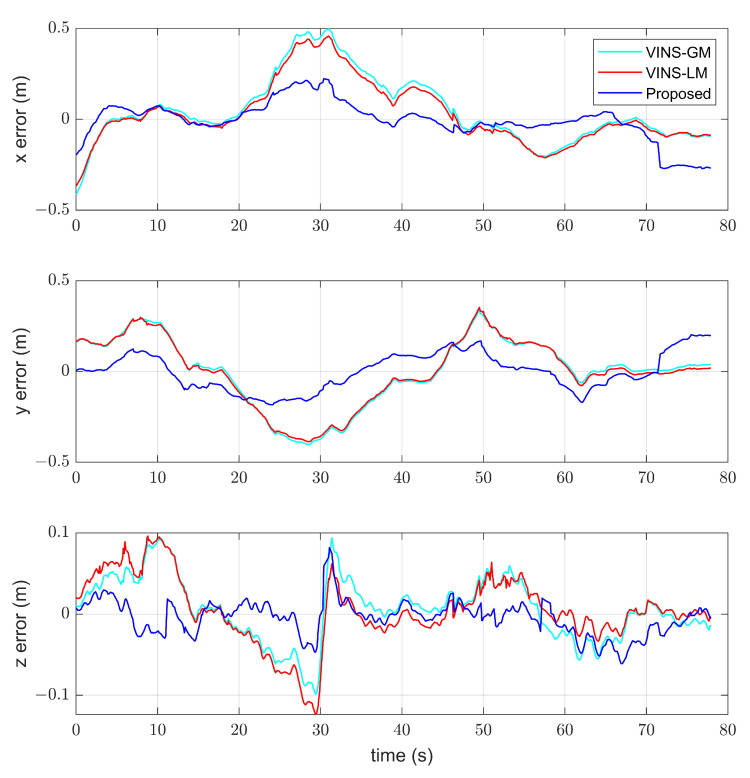
Position errors of the proposed method compared with VINS-LM and VINS-GM in MH05.

In order to demonstrate the consistency of the proposed algorithm, we plotted the position errors of the proposed approach in comparison with those of VINS-LM and VINS-GM, using the MH05 sequence as an example. As the proposed method conducts online estimation of the transformation between the local reference frame and global map reference frame, the results demonstrate an improved consistency. Specifically, as depicted in Figure 7, our algorithm achieves a superior consistency compared to the other two methods.

**Figure 8:**
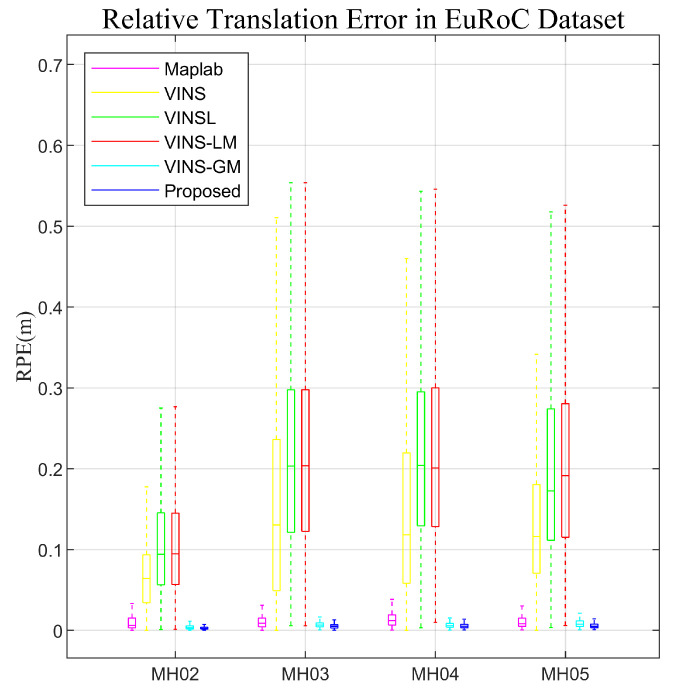
Relative translation error in MH02.

Figure 8 presents the relative pose error in the EuRoC dataset. As seen in the figure, the use of global-map-based measurements leads to a substantial reduction in the relative translation error, which is an order of magnitude lower than other algorithms. Additionally, compared to VINS-GM, the proposed method yields a smaller relative translation error. However, the effect is smaller compared with the improvement achieved using global-map-based measurements. This result is expected since whether or not to optimize the augmented state ${}^{G}T_{L}$ has little effect on the relative pose in a short term.

### 5.2. 4Seasons Dataset

**Figure 9:**
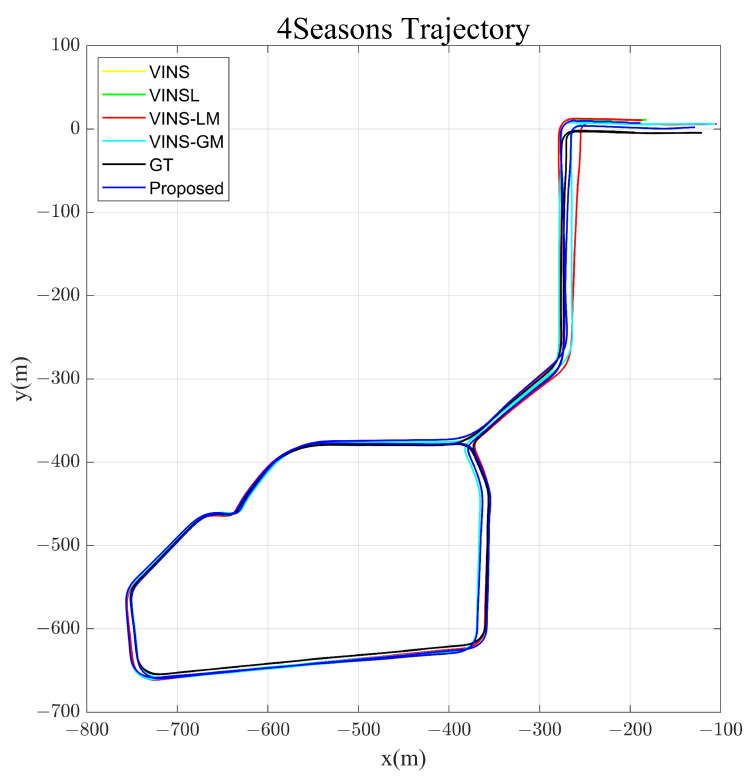
Trajectories of different methods in 4Seasons dataset. VINS, VINSL, VINS-LM, VINS-GM and the proposed algorithm were tested to compare with the ground truth (GT). GT is provided by the extended stereo direct sparse visual odometry integrating IMU measurements, camera measurements and RTK-GNSS measurements.

For the 4Seasons dataset, we also compared the errors of the results of different algorithms, as shown in Table 1. Figure 9 shows the trajectories of the different algorithms. Due to the trajectories being aligned to the ground truth, there are slight differences in the starting points of different algorithms. However, this does not affect the fact that the timestamps of the various starting points are the same. As is seen from the results, the filter-based framework Maplab fails to build a map, because its VIO quickly drifts far away on the dataset. In contrast, the proposed algorithm achieves the smallest ATE among all algorithms. This favorable outcome can be attributed to the application of the global map and online estimation of ${}^{G}T_{L}$.

### 5.3. Beiqing Road Dataset

**Figure 10:**
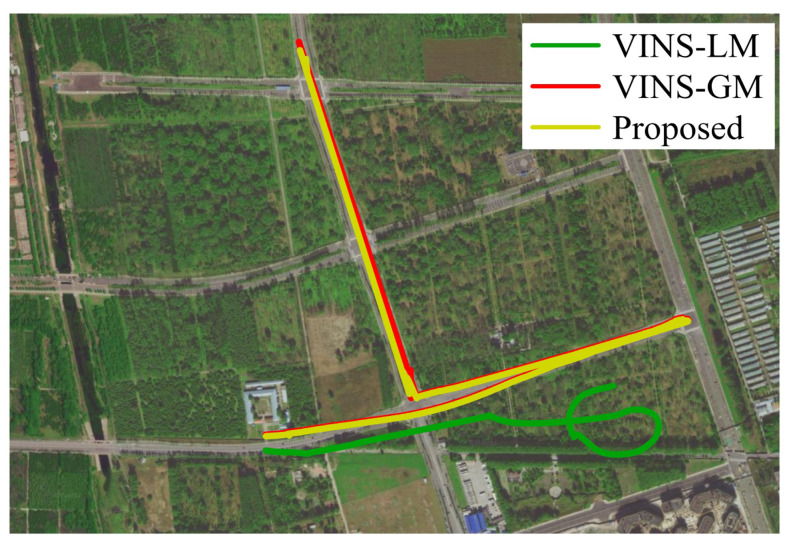
Trajectories of different methods in Beiqing Road dataset. The yellow line is the trajectory estimated by the proposed method. The green line is the trajectory estimated using VINS-LM.

The Beiqing Road dataset consists of two sequences. We initially constructed and stored the map of the first sequence, followed by map loading for algorithm validation on the second sequence. As our GNSS positioning mode utilizes a single point, there are no quantitative experimental results to present. Instead, we conduct a qualitative assessment to demonstrate the advantages of a global map over a local map. In this experiment, we test VINS-LM, VINS-GM and the proposed algorithm. The result on the Beiqing Road dataset is shown in Figure 10. The yellow line is the trajectory of the result from the proposed algorithm. The green line is the trajectory of the result from the VIO pipeline with a local map (VINS-LM). The red line is the trajectory of the result from VINS-GM. In the absence of global measurements, the pre-built map is incorrect, and consequently, erroneous information is propagated leading to trajectory drift. To visually illustrate this issue, we have aligned the complete trajectory with the Baidu Map, as illustrated in Figure 10. Obviously, the alignment results attest to the effectiveness of the global map.

## 6. Conclusions and Discussion

In this paper, we propose a two-stage global drift-free map-based localization scheme that fuses global-map-based measurements with the local pose estimation from a VIO pipeline to obtain accurate global poses. Autonomous vehicle localization is achieved by fusing VIO with map-based localization within the global reference map. We conducted experiments using various datasets and compared the results with those obtained from other methods. From the experimental results, we can draw these conclusions:

- Incorporating pre-built maps can provide additional constraints in the VIO localization process, leading to improved accuracy. The experimental results show that map-based localization methods, including VINS-LM, VINS-GM and the proposed method, generally outperform pure VIO methods.
- The use of global pre-built maps is more effective in bounding the drift of VIO than that of local pre-built maps, as the global pre-built maps are more reliable. The experimental results from the Beiqing Road dataset demonstrate the advantages of using global pre-built maps.
- The online estimation of the transformation between the global map frame and the local VIO frame, instead of treating it as a constant, can improve the accuracy and consistency of localization.

To ensure the reliable performance of map-based localization methods, the accuracy of the map is crucial as it can impact the localization process. The incorporation of global localization information can enhance the reliability of the map. In this paper, we specifically focus on the incorporation of GNSS information into maps. Moreover, various other sources of global localization information can be utilized, such as WiFi, motion capture systems and ultra-wide-band localization systems. It is worth noting that incorporating global localization information instead of local information into maps enhances the map’s ability to compensate for VIO drift.

It should be noted that the proposed method is well-suited for tasks that are repeatedly performed in the same area. This allows for the effective utilization of map information to improve the localization performance. However, even in unknown environments without pre-built maps, our method can still perform localization through VIO.

Moreover, it is crucial to successfully associate the captured images in the localization process with the pre-built map. Therefore, in the future, we plan to improve the algorithm to achieve a better recall rate on loop detection in more challenging environments. Specifically, we aim to employ a more robust matching method to enhance our map-based localization approach.

## Figures and Tables

**Table 1:** RMSE (m) of absolute trajectory error for different methods on different datasets with the best performance numbers highlighted in bold fonts.

Dataset	EuRoC				4Seasons
**Sequence**	**MH02**	**MH03**	**MH04**	**MH05**	**2020-03-24-17-45-31**
Maplab [10]	0.110	0.182	0.603	0.627	-
VINS	0.092	0.167	0.203	0.340	15.313
VINSL	0.039	0.069	0.112	0.183	14.156
VINS-LM	0.073	0.068	0.110	0.259	13.457
VINS-GM	**0.029**	0.142	0.138	0.273	11.428
Proposed	0.034	**0.059**	**0.102**	**0.136**	**8.724**

## Data Availability

The data underlying the results presented in this paper are not publicly available at this time but may be obtained from the authors upon reasonable request.

## Abbreviations

The following abbreviations are used in this manuscript:

VIO	Vision–Inertial Odometry
IMU	Inertial Measurement Unit
SLAM	Simultaneous Localization and Mapping
GNSS	Global Navigation Satellite System
EKF	Extended Kalman Filter
UAV	Unmanned Aerial Vehicle
BoW	Bag-of-Words
PnP	Perspective-n-Point
VINS	Visual–Inertial System
VINSL	Visual–Inertial System with Loop Closure
VINS-LM	Visual–Inertial System with Local Map
VINS-GM	Visual–Inertial System with Global Map
RMSE	Root Mean Square Error
ATE	Absolute Trajectory Error
